# Effect of rollover footwear on metabolic cost of ambulation, lower limb kinematics, kinetics, and EMG related muscle activity during walking

**DOI:** 10.1186/1757-1146-5-S1-O4

**Published:** 2012-04-10

**Authors:** Saeed Forghany, Christopher Nester, Barry Richards, Anna Hatton

**Affiliations:** 1 School of Health Sciences, University of Salford, UK; 2 Musculoskeletal Research Centre, School of Rehabilitation Sciences, Isfahan University of Medical Sciences, Iran

## Background

Footwear with a curved sole profile shifts the point of contact between the shoe and floor anteriorly compared to a flat shoe. There have been various reports of changes in ground reaction forces, external joint moments, and (rather inconclusively) muscle activity and energy consumption during walking in this “rollover” footwear [1-6]. The aim of this study was to investigate the effect of two types of rollover footwear (one a new prototype) on walking speed, metabolic cost of gait, lower limb kinematics, kinetics and EMG muscle activity.

## Methods

Twenty subjects walked in four footwear conditions: (1) a flat control shoe; (2) a flat control shoe weighted to match the mass of the new rollover shoe prototype; (3) the new prototype rollover shoe; (4) a MBT shoe. Data relating to metabolic energy and temporal aspects of gait were collected during a 6 minute walk test. Data on the lower limb kinematics, joint moments, muscle activity and foot pressure were collected during walking on a straight 10 metre course.

## Results

The curved sole moved the contact point under the shoe anteriorly during early stance. This altered ankle moments and reduced ankle plantarflexion after initial contact. In mid stance the roll over footwear reduced ankle dorsiflexion and overall the ankle moved less in roll over footwear. Ground reaction force loading rates were increased by the rollover footwear. There were notable elevations in early stance calf EMG activity. Effects on energy cost of ambulation were negligible. Rollover footwear reduced plantar pressures under the heel and forefoot, and redistributed pressure towards the midfoot. Shoe weight had no effect on any parameters.

## Conclusion

Rollover footwear is able to modify ankle kinematics and moments and the activity of some ankle musculature. Effects proximal to the leg were small. There were no effects on the temporal or energy aspects of gait.
